# Chitosan-Coated Alginate Microcapsules Loaded with Herbal galactagogue Extract: Formulation Optimization and Characterization

**DOI:** 10.22037/ijpr.2019.1100776

**Published:** 2019

**Authors:** Nasim Khorshidian, Arash Mahboubi, Naser Kalantari, Hedayat Hosseini, Mojtaba Yousefi, Masoumeh Arab, Adriano Gomez da Cruz, Amir Mohammad Mortazavian, Fatemeh Sadat Mahdavi

**Affiliations:** a *Student Research Committee, Department of Food Technology, Faculty of Nutrition Sciences and Food Technology/National Nutrition and Food Technology Research Institute, Shahid Beheshti University of Medical Sciences, Tehran, Iran. *; b *Food Safety Research Center (Salt), Semnan University of Medical Sciences, Semnan, Iran.*; c *Department of Pharmaceutics, School of Pharmacy, Shahid Beheshti University of Medical Sciences, Tehran, Iran. *; d *Department of Community Nutrition, School of Nutrition and Food Sciences, Shahid Beheshti University of Medical Sciences, Tehran, Iran. *; e *Department of Food Technology, Faculty of Nutrition Sciences and Food Technology/National Nutrition and Food Technology Research Institute, Shahid Beheshti University of Medical Sciences, Tehran, Iran.*; f *Department of Food Science and Technology, Federal Institute of Education of Rio de Janeiro, Maracan˜a, Rio de Janeiro, Brazil.*; g *Food Safety Research Center, Shahid Beheshti University of Medical Sciences, Tehran, Iran. *; h *Student Research Committee, Alborz University of Medical Sciences, Karaj, Iran. *

**Keywords:** Galactagogues, Herbal extract, Microencapsulation, Sodium alginate, Chitosan, External gelation

## Abstract

Many herbs and spices have been recommended traditionally as galactagogues and several commercial formulations prepared using herbs. Due to the presence of various compounds such as polyphenols, flavonoids, isoflavones, and terpenes, bitter and stringent taste is elicited that make the consumption of these herbal preparations unpleasant. Moreover, these compounds are unstable when exposed to environmental conditions. In this regard, different approaches are used for taste masking such as microencapsulation. In the present study, microcapsules containing herbal galactagogue extract were developed through emulsification/external gelation and Box-Behnken design was used to investigate the effects of independent variables (sodium alginate: 1-1.5%, calcium chloride: 0.2-1% and extract concentrations: 1-5%) on encapsulation efficiency (EE%). Following evaluation of the model, the optimum condition of encapsulation process was selected as 1.49% sodium alginate, 0.84 CaCl_2_, and 1.58% extract with EE% of 77.97%. Microcapsules had an acceptable spherical morphology and the results of Fourier transform-infrared spectroscopy (FTIR) revealed the presence of the extract within the microcapsules. The mean diameters of the uncoated and chitosan-coated microcapsules were 52 and 123 μm and encapsulation yield was 50.21 and 69.7%, respectively. The polydispersity index of 0.45 and 0.48 were an indicative of polydisperse nature of the microcapsules. The loss of flavonoids in microcapsules stored at two different temperatures was insignificant. The *in-vitro* release in simulated gastric fluid (SGF; pH 1.2) and simulated intestinal fluid (SIF; pH 7.4) were 48.1% and 80.11%, respectively during 24 h. The prepared extract-loaded microcapsules have potential to be used in matrices with neutral pH.

## Introduction

Galactagogues are pharmaceutical agents, foods, or herbal supplements used to support the initiation, continuation, or augmentation of breast milk production (1). Herbs and natural substances have been used traditionally since ancient times in order to stimulate milk production. The most frequently used herbs in commercial preparations and formulations are shatavari, torbangun, milk thistle, fenugreek, fennel, caraway, dill, cumin, and anise (2, 3). The main active components in these plants that contribute to galactapoetic effect are polyphenols, flavonoids, isoflavones, and terpenes (4) which elicit bitter and astringent taste in the derived extracts and therefore, consumption of the herbal preparations by breastfeeding women is aversive. In general, bitter-tasting products are unpleasant for consumers leading to noncompliance and thus decreased therapeutic efficacy. It has been proved that consumers prefer the products presenting health benefits without compromising flavor, taste, and color (5, 6). In this respect, different approaches for taste masking of pharmaceutical formulations have been proposed such as utilization of sweeteners and flavoring agents, granulation, ion exchange resins, solid dispersion system, mass extrusion method and microencapsulation (7, 8). Furthermore, polyphenols have poor stability and are usually affected by pH variation, light, temperature, oxygen, and presence of metal ions (9). 

In this regard, microencapsulation is a technique that entraps active ingredients in polymeric materials and creates a physical barrier against environmental conditions as well as sustained release and masking of unpleasant taste (10, 11). Encapsulation process can be carried out through different approaches and polymeric substances as well as materials (12, 13). Ionotropic gelation of alginate with calcium chloride and formation of calcium alginate gel beads have been extensively used in encapsulation of different bioactive ingredients (14-16). Alginate is a water-soluble polyanionic polymer composed of 1, 4-linked-α-L-guluronic acid and β-D-mannuronic acid residues derived from brown see-weeds and marine algae (17, 18). Although the biocompatibility, biodegradability, immunogenecity, and non-toxicity of alginate have made it an excellent carrier, the macroporous structure of calcium alginate beads leads to rapid dissolution of microcapsules, sudden release of core substances, and low encapsulation efficiency (19). In order to increase the stability and improve the permeability, chitosan has been extensively employed as a coating for calcium alginate beads which forms a strong complex membrane via electrostatic interaction between its amino residues and the carboxyl residues present in alginate (20, 21). To the best of our knowledge, no study has been conducted to encapsulate herbal galactagogue extract in alginate microcapsules, thus this study was designed to develop and characterize microcapsules containing mixture of herbal galactagogue extract using W/O emulsification followed by external gelation which can be used in matrices with neutral pH and formulation of food products with galactapoetic property. RSM in combination with Box-Behnken was applied to explore the effects of three independent variables (sodium alginate, calcium chloride and extract concentrations) on encapsulation efficiency as well as to develop a mathematical model for prediction and determination of optimum conditions for the maximum encapsulation efficiency. 

## Experimental

*Materials*

Sodium alginate (molecular mass of 1.97×10^5^ Da, mannuronate/guluronate ratio = 0.6) was from BDH (Poole, UK). Medium-molecular weight chitosan (deacetylation degree 75-85%), quercetin (≥ 95%), calcium chloride and Span 80 were purchased from Sigma-Aldrich (St. Louis, MO, USA). Herbal galactagogue extract (containing *Foeniculum vulgare*, *Cuminum cyminum*, *Trigonella foenum* and *Anethum graveolens* extracts) was purchased from Goldaru Co. (Isfahan, Iran). Sunflower oil with no added antioxidant was supplied by Ladan (Tehran, Iran). 

Aluminum chloride and potassium acetate were purchased from Merck (Darmstadt, Germany). 

*Preparation of chitosan-coated alginate microcapsules containing herbal galactagogue extract*

Microcapsules were prepared by W/O emulsion technique followed by external gelation. Alginate solution at different concentration was prepared in distilled water stirred by magnetic stirrer overnight. Herbal galactagogue extract was added to alginate solution and mixed completely. Afterwards, the W/O emulsion was prepared by blending alginate solution with sunflower oil (containing 1% v/v span 80) using an ultra-Turrax homogenizer (IKA T25- Digital Ultra Turrax, Staufen, Germany) at a speed of 8000 rpm for 10 min. Then, microcapsules were formed by adding CaCl_2_ dropwise to emulsion under stirring at 400 rpm. Stirring was continued for 45 min. Then, the microcapsules were recovered by centrifugation at 1800 rpm for 5 min followed by vacuum filtration. Microcapsules were washed severally with deionized water containing Tween 80. The obtained microcapsules were immersed in chitosan solution with pH of 4.5 (1% w/v in 1% v/v acetic acid) under stirring for 15 min followed by centrifugation at 2000 rpm for 3 min and washing with distilled water. Finally, the beads were frozen at -80°C for 2 h and lyophilized with a freeze drier for 14 h (ALPHA 2-4; Christ, Harz, Germany). 

*Formulation optimization of chitosan-coated alginate microcapsules loaded with herbal galactagogue extract*

In order to optimize the conditions for the preparation of chitosan-coated alginate microcapsules, Design-Expert software (V.7.0.0, Stat-Ease, Inc., Minneapolis, USA) with Box-Behnken response surface methodology was used to determine relationships among the variables and responses. Three independent variables were chosen as sodium alginate concentration (X_1_), herbal galactagogue extract concentration (X_2_), and CaCl_2_ (X_3_) while the dependent variable was encapsulation efficiency (EE %). The coded levels and actual values of the independent variables are presented in Table 1. A set of 17 experiments were employed with five replicates (used to estimate experimental error) of the center point (Table 2). For the prediction of optimal point, a quadratic model was fitted to correlate relationship between independent variables and the response, which accounts for variations caused by linear and quadratic order effects as well as by interactions. For the factors, the equation is: 

Equ. 1 Y=β0+∑i=1kβiXi+∑i=1kβiiXj2+∑i˂j∑4kβij XiXj+ε

Where Y is the dependent variable (EE%); *Xi* and *Xj* are levels of independent variables; *β*_0_ is a constant; *βi*, *βii,* and *βij* are interaction coefficients of linear, quadratic, and interaction coefficients, respectively; k is the number of independent parameters (k = 3 in this study); and *ε *is the error (22). Following determination and validation of the model, optimum formulation regarding to encapsulation efficiency was selected for preparation of chitosan-coated alginate microcapsules containing herbal galactagogue extract and the microcapsules were assessed in respect of physicochemical characteristics and release properties. 

*Encapsulation efficiency (EE%) of herbal galactogogue extract*

In order to determine the EE%, accurately weighed amounts (200 mg) of microcapsules were suspended in 30 mL phosphate buffer (pH 7.4) and stirred at 150 rpm at room temperature until the complete dissolution of microcapsules. Then, the solution was centrifuged at 9000 rpm for 5 min and the content of total flavonoid was determined in the supernatant. EE% was calculated using the following equation:

Equ. 2 $$\mathrm{EE}(\%)=\frac{\mathrm{Total amount of loaded flavonoid}}{\mathrm{Initial amount of flavonoid}}\times 100$$

*Quantifications of the flavonoids content*

Total flavonoid content was determined using the aluminum chloride colorimetric method (23). In brief, 0.5 mL of sample was mixed with 2.8 mL of distilled water, 1.5 mL of ethanol, 0.1 mL of 10% aluminum chloride solution and 0.1 mL of 1 mol/L potassium acetate. 

The mixture was kept for 30 min and the absorbance of the samples was measured in a spectrophotometer (OPTIMA SP-3000 plus, Tokyo, Japan), at 415 nm. Quercetin was used as the standard in the range of 1-300 μg/mL. 

*Encapsulation yield (EY%)*

The encapsulation yield was calculated as the ratio of the mass of microcapsules obtained at the end of the process to the mass of raw materials used in preparation of chitosan-coated and uncoated according to the following equation:

Equ. 3 $$\mathrm{EY\%}=\frac{\mathrm{Mass of microcapsules}}{\mathrm{Mass of raw materials}}\times 100$$

*Morphological characterization of microcapsules*

The morphology of chitosan-coated microcapsules prepared at optimum condition, was examined using a scanning electron microscope (SEM) (Philips XL30). 

The samples were mounted to the specimen holder with a double-sided adhesive tape and vacuum coated with gold. SEM photographs were taken at the required magnification at room temperature and examined using an acceleration voltage of 25 kV.

*Particle size analysis*

The particle size distribution and the mean particle size of the samples were determined by dynamic light scattering using Malvern Nano ZS (red badge) ZEN 3600. Analyses were carried out using aqueous dispersions of loaded microcapsules in triplicate. 

*Fourier transform infrared (FTIR) spectroscopy*

FTIR spectra of pure herbal galactagogue extract, sodium alginate, chitosan, chitosan-coated alginate beads without extract and chitosan-coated alginate beads with extract were obtained using an FTIR spectrophotometer (PerkinElmer Spectrum RX I, Waltham, MA, USA). 

The samples were mixed with KBr and compressed to form discs. Each sample was scanned 16 times in the range of 4000-450 cm^ -1^ at a resolution of 4 cm^-1^. Data were analyzed by Spectrum V 5.0.1 software (PerkinElmer, Waltham, MA, USA).

*Stability studies*

To evaluate the stability of flavonoids in microcapsules, 5 gr of freeze-dried chitosan-coated (prepared at optimum condition) microcapsule was placed in 40-mL bottles, tightly capped and stored away from light either at refrigerator temperature (4 °C) or room temperature (25 °C) for 120 days. The content of total flavonoids in the samples was determined after 0, 30, 60, 90, and 120 days of storage according to the method described in the total flavonoid quantification section. The results were reported as the percent loss of total flavonoids content.

*In-vitro release studies*

*In-vitro* release of extract from chitosan-coated microcapsules was investigated in simulated gastric fluid (SGF) and simulated intestinal fluid (SIF) during 24 h as follows: approximately 300 mg of microcapsules were suspended in 100 mL solution and maintained at 37 °C at 100 rpm. 

The solutions were 0.1 mol/L HCL (pH 1.5) and phosphate buffer saline (PBS, pH 7). At predetermined time intervals, the samples (5 mL) were collected from release medium and replaced with fresh solutions. Aliquots (5 mL) were centrifuged at 9000 rpm for 10 min and the supernatant was analyzed for flavonoid concentration. All experiments were carried out in triplicate and percentage of cumulative amount of released extract was plotted against time. 

*Statistical analysis*

Data analysis was performed using Design Expert software (V. 7, Stat-Ease, Inc., Minneapolis, USA) with appropriate ANOVA studies, used for mathematical modeling in response surface methodology. 

## Results and Discussion

*Model evaluation*

The encapsulation efficiencies obtained in different experiments are presented in Table 2. The quadratic model was selected as the best-fitting model having an insignificant lack of fit (0.1358) and maximum value of the adjusted R-square and the predicted R-square. 

The high value of R^2^ (0.9851) and R^2^ adjusted (0.9659) is an indicative of agreement between experimental results and the data predicted by the model. In order to find the significant parameters affecting the encapsulation efficiency, analysis of variance (ANOVA) was performed. The results are presented in Table 3. 

**Table 1:** Independent variables and their levels used in Box-Behnken design

**Independent variables**	**Symbol**	**Coded levels**		
		**-1**	**0**	**1**
Sodium alginate%	X1	1	1.25	1.5
Herbal galactagogue extract%	X2	1	3	5
CaCl2	X3	0.2	0.6	1

**Table 2:** Box-Behnken design and effect of independent variables on encapsulation efficiency as a response

**Runs**	**Sodium alginate (%)**	**Herbal galactagogue extract (%)**	**CaCl** **2 ** **(M)**	**Actual Encapsulation efficiency (%)**	**Predicted Encapsulation efficiency (%)**
1	1.25	5	0.2	37.61	35.62
2	1.25	3	0.6	62.29	63.54
3	1.25	3	0.6	64.76	63.54
4	1.25	1	1	68.15	70.14
5	1	5	0.6	35.5	34.83
6	1	3	0.2	50.09	52.75
7	1	1	0.6	53.2	51.79
8	1.25	3	0.6	65.1	63.54
9	1.5	3	0.2	56.54	57.12
10	1.25	1	0.2	61.12	59.87
11	1	3	1	52.15	51.57
12	1.5	5	0.6	32.18	33.59
13	1.25	3	0.6	61.11	63.54
14	1.5	1	0.6	74.22	74.89
15	1.25	3	0.6	64.42	63.54
16	1.5	3	1	71.73	69.07
17	1.25	5	1	34.89	36.14

**Table 3:** Analysis of variance for response quadratic model

Encapsulation efficiency (%)					
**Source**	**Sum of Square**	df*	**Mean Square**	**F-value**	**Prob > F**
Model	2833.00	9	314.78	51.40	< 0.0001**
X1	239.04	1	239.04	39.03	0.0004**
X2	1696.82	1	1696.82	277.06	< 0.0001**
X3	58.10	1	58.10	9.49	0.0178**
X1X2	148.11	1	148.11	24.18	0.0017**
X1X3	43.10	1	43.10	7.04	0.0328**
X2X3	23.77	1	23.77	3.88	0.0895
X1 2	60.42	1	60.42	9.86	0.0164**
X2 2	506.98	1	506.98	82.78	< 0.0001**
X3 2	18.93	1	18.93	3.09	0.1221
Residual	42.87	7	6.12		
Lack of Fit	30.71	3	10.24	3.37	0.1358
Pure Error	12.16	4	3.04		

* Degree of freedom.

** Significant at 0.05 level.

**Figure 1:**
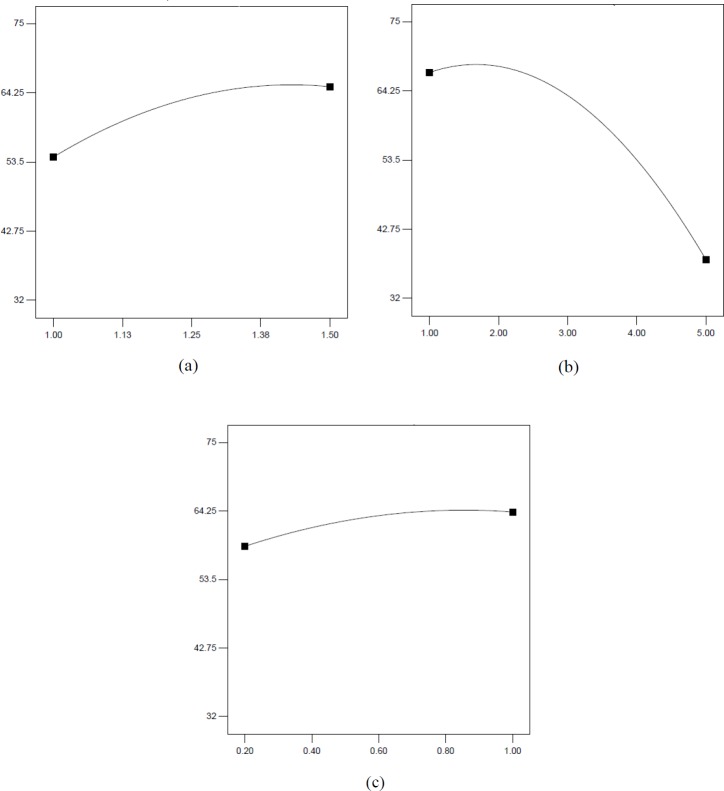
Effect of sodium alginate (a), extract (b) and CaCl2 (c) concentration on encapsulation efficiency

**Figure 2:**
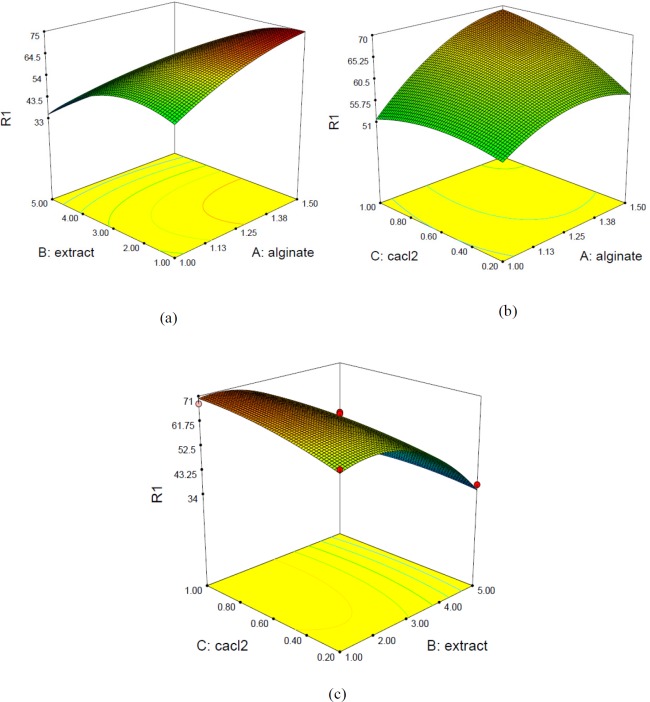
3D surface plots for EE% with respect to sodium alginate and extract (a), CaCl2 and sodium alginate (b) and extract and CaCl2 (c)

**Figure 3:**
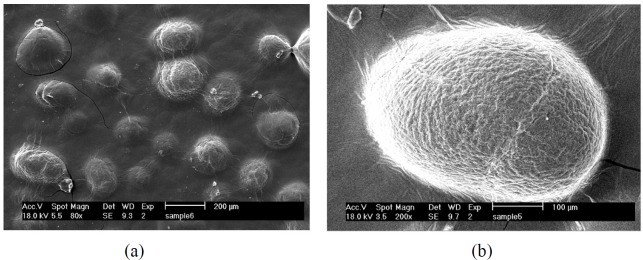
SEM images of chitosan-coated microcapsules loaded with extract; 80× (a) and b) 200× (b)

**Figure 4:**
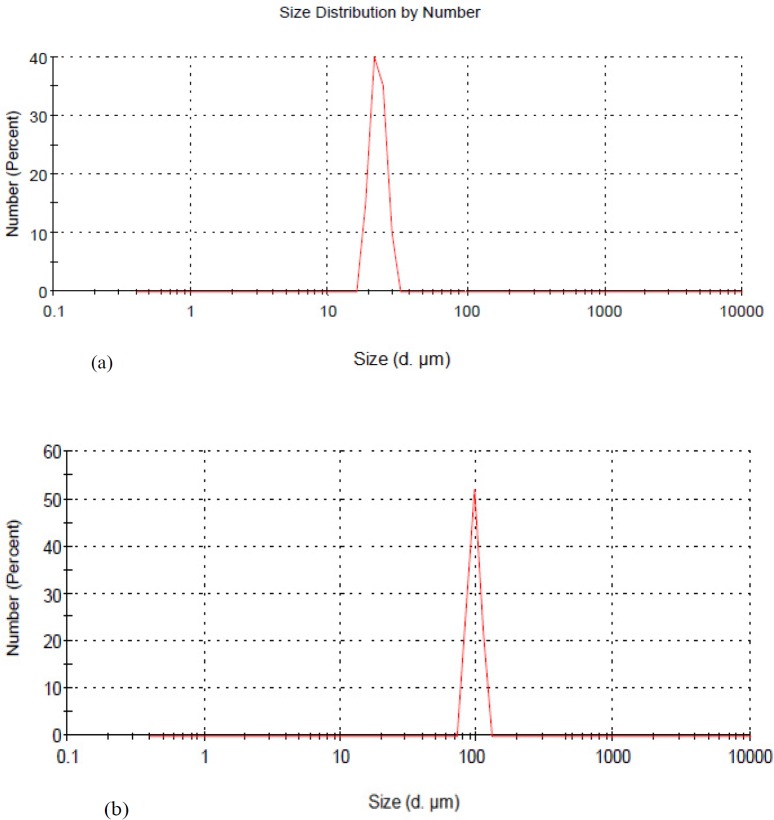
Size distribution by number for uncoated alginate microcapsules loaded with extract (a) and chitosan-coated alginate microcapsules loaded with extract (b)

**Figure 5:**
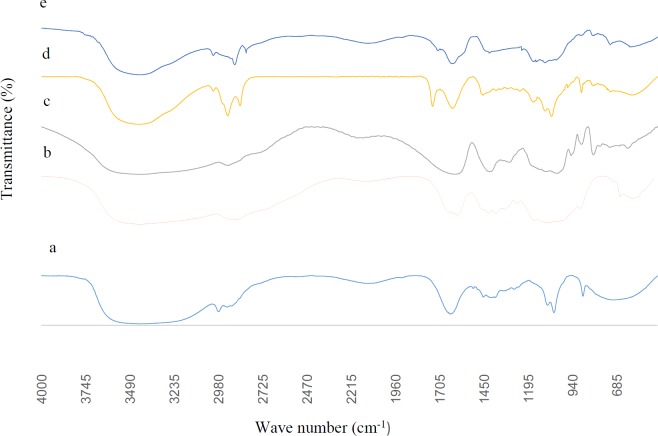
FTIR spectra of herbal galactagogue extract (a), chitosan (b), sodium alginate (c), chitosan-coated alginate microcapsules containing extract (d) and blank chitosan-coated alginate microcapsules (e)

**Figure 6:**
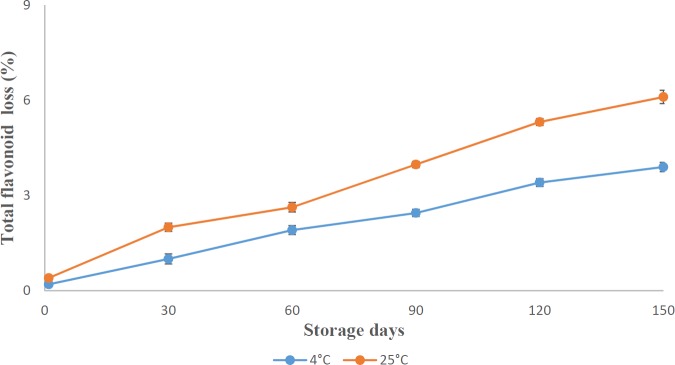
Total flavonoid loss (%) in chitosan-coated microcapsules stored at 4 °C and 25 °C during storage

**Figure 7:**
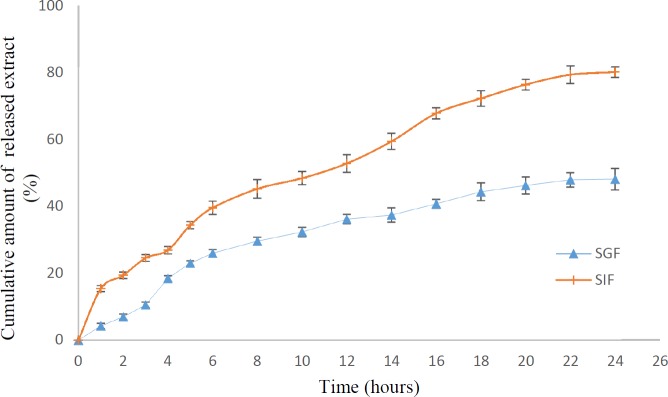
*In-vitro *cumulative release of herbal galactagogue extract from microcapsules in SGF and SIF

The significance of each coefficient was specified by p-value. The values less than 0.05 indicate significant variables. Among the tested variables in this study, X_1_ (sodium alginate concentration), X_2_ (extract concentration), X_3_ (CaCl_2_ concentration), (X_1_)^2^ (sodium alginate concentration × sodium alginate concentration), (X_2_)^2^ (extract concentration × extract concentration), X_1_X_2_ (sodium alginate concentration × extract concentration) and X_1_X_3_ (sodium alginate × CaCl_2_ concentration) were significant terms of the model. 

The coefficients of independent variables are given in equation as below:

Y = -96.6556 + 190.2000 X_1_ + 26.2182 X_2_ – 9.2493 X_3_- 12.1700 X_1_X_2_ + 32.8250 X_1_X_3 _– 60.6080 X_1_^2^- 2.7432 X_2_^2^

The equation shows that the sodium alginate and extract influenced EE% in a quadratic and linear model while CaCl_2 _had linear effect. Figure 1 illustrates linear effects of sodium alginate, extract and CaCl_2 _on EE%. Figure 2 illustrates the response surface plots for EE% with respect to sodium alginate and extract (a) and CaCl_2_ and sodium alginate (b). 

*Formulation optimization*

The optimum point with a maximum predicated value of EE% (78.028 %) was obtained at alginate concentration of 1.49 %, extract concentration of 1.58 % and CaCl_2_ level of 0.84 %. The accuracy of predicated condition was examined by performing independent experiments at the optimal point. The results revealed that the predicated value from the model was reasonably close to observed value (77.97%).

*Effect of microencapsulation conditions on encapsulation efficiency*

Figure 1 illustrates linear effect of independent variables on EE%. As shown, encapsulation efficiency was increased by increasing sodium alginate concentration from 1 to 1.5% and extract concentration up to 2%. Increasing CaCl_2_ concentration enhanced EE% and was constant in the range of 0.8-1%. The simultaneous effects of two factors on response while keeping the other variable constant at its middle level (center value of testing ranges) are presented in forms of 3D surface plots in Figure 2. The tortuous surface shows a strong interaction between two factors. Figure 2a shows the 3D surface plot for combined effects of sodium alginate and galactagogue extract concentration (X_1_X_2_) on EE%. A higher encapsulation efficiency was obtained with extract concentration of 1-2% and sodium alginate concentration of 1.5%, but when extract level was enhanced, EE% decreased even at high concentration of sodium alginate. EE% increase by enhancing alginate level might be due to the formation of a more compact membrane which inhibits leakage of extract to external solution (24). Similarly, Chan (15) stated that EE% of a model oil in Ca-alginate beads increased from 60% for the 5 g/L alginate solution to 90% for the 25 g/L solution. The increase of EE% by enhancement of alginate concentration was confirmed in the study of Lotfipour *et al*. (25) who pointed that alginate concentration was the most affective factor on EE% of *Lactobacillus acidophilus* and also an increase in EE% of propranolol incorporated into calcium alginate beads (26). In a study conducted by Soliman *et al*. (27), EE% was decreased with increasing the alginate concentration over 2% due to formation of pores with smaller size and consequently a lower amount of ingredient can be entrapped within the polymer matrix. Calcium ions interact preferentially with guluronic sequences of sodium alginate and hydrogel networks are formed. Therefore, it can be assumed that sodium alginate with high guluronic acid denoted as high G (the alginate used in this study), are more susceptible to CaCl_2_ level. 

By increasing extract level, the EE% was reduced. Similarly, Zam *et al*. (28) stated that the highest loading efficiency of pomegranate polyphenols in alginate beads was obtained by 1% extract. Extract is water soluble and the amount of encapsulated extract is dependent on the water amount of hydrogel. Therefore, applying high concentrations of lemon balm extract did not increase the encapsulation efficiency (24). Moreover, it can be explained by the saturation of extract loading into alginate microcapsules (29). 

Figure 2b presents the 3D surface plot for combined effects of sodium alginate and CaCl_2_ concentration (X_1_X_3_) on EE%. Increasing the level of CaCl_2 _particularly in the range of 0.6-0.8% and simultaneously increase of sodium alginate concentration led to the highest EE%. Increasing the level of Ca^+2^ causes rapid cold setting and a densely cross-linked gel with lower porous structure; therefore, lower amount of herbal galactagogue extract can be entrapped in gel matrix and subsequently EE% would decrease (27). These results are in agreement with the study conducted by Najafi-Soulari *et al*. (24) who pointed that encapsulation efficiency of lemon balm extract decreased by increasing CaCl_2_ concentration up to 1% and became constant at higher concentrations. Consistently, it was reported that by increasing the level of CaCl_2_, more Ca^+2^ ions diffuse into the drug-loaded alginate beads and more drug would displace from alginate matrix by Ca^+2^ ions leading to a decrease in EE% (26). 

Figure 2c shows combined effects of extract and CaCl_2 _on EE%. By increasing extract concentration in the range of 1-2% and CaCl_2 _in the range of 0.8-1%, EE% increased. Although, this increasing trend was higher at higher concentration of CaCl_2_, the difference was not remarkable. Therefore, the interaction of these two factors was not statistically significant (*p* ˃ 0.05).

*Encapsulation yield*

The microencapsulation yield (EY%) is important from the economic point of view in any encapsulation process, considering the cost of polymers and active principles used (30). EY% calculated in uncoated and chitosan-coated microcapsules was 50.21 ± 0.8 % and 69.7 ± 0.5 %, respectively. In a study by Samakradhamrongthai *et al*. (31), the encapsulation yield of freeze-dried *Michelia alba* D.C. extract was reported 50.01%. In another study, EY% of freeze-dried alginate microcapsules containing lactobacilli was 50.5 (30). Sabitha *et al*. (32) obtained EY% in the range of 55-68% for different antitubercular drugs encapsulated in chitosan-alginate matrix. In chitosan-coated microcapsules, due to an extra coating and increase in the thickness of the microcapsule wall, EY% was higher. It was reported that the higher amount of wall material available for formation of a thicker microcapsule wall resulted in increasing product recovery (33). Furthermore, using chitosan as a coating would increase the total solid content of feed solution in freeze drying process (34). 

*Morphology of microcapsules*

The morphology of chitosan-coated microcapsules prepared at optimum condition was studied by SEM as shown in Figure 3 As can be seen, an acceptable spherical morphology was obtained. The absence of ideal spherical morphology and rough structure can be probably attributed to the drying process that causes certain invaginations in the particles (35). 

Similarly, Saikia *et al*. (36) pointed out that freeze-dried encapsulates of polyphenol powder were irregular in the shape compared to spray-dried microcapsules. Dai *et al*. (37) reported a rough surface in alginate-chitosan hydrogel beads with cracks and wrinkles due to partial collapsing of the polymer network during dehydration. Insufficiency of ideal spherical structure and smoothness was also reported in other studies (21, 38).

*Mean particle size and particle size distribution of microcapsules*

Figure 4 presents particle size distribution of chitosan-coated and uncoated microcapsules. Diameter of uncoated and chitosan-coated microcapsules were in the range of 46-75 μm and 90-150 μm and mean diameter of 52 ± 0.76 μm and 123 ± 2.3 μm, respectively. It has been reported that microcapsules smaller than 200 μm show a more prolonged passage time and the possibility of controlled release of the encapsulated drug substance (38, 39).

As evident in Figures 4, the microcapsules had a narrow size distribution. The polydispersity index (PDI) values of 0.48 and 0.45 for uncoated and chitosan-coated alginate microcapsules confirm acceptable polydisperse nature of microcapsules in aqueous. Coating of alginate microcapsules with chitosan increased the particle size. This is in agreement with the results obtained by Nualkaekul *et al*. (40) that reported an increase in particle size of alginate beads loaded with *Lactobacillus plantarum* by chitosan coating. Furthermore, loading of extract in microcapsules was affective on particle size increment. Similarly, Khaksar *et al*. (41) stated an increase of particle size by entrapment of nisin in alginate-high methoxy pectin microparticles. Hui *et al*. (42) also reported an increase in microcapsules’ size loaded with PentaHerbs extract compared to unloaded microcapsules.

*FTIR analysis*

The functional groups and intermolecular interactions within the microcapsules were investigated by FTIR. Figure 5 represents FTIR spectrum of herbal galactagogue extract, chitosan, sodium alginate, and chitosan-coated alginate microcapsules containing extract. Figure. 5a shows the spectrum of galactagogue extract band at 3419, 2979, 1645, 1411, 1047, and 713 cm^-1^ that can be assigned to OH stretching, sp^3^ C-H stretching, C=O stretching, α-CH_2_ bending, C–O stretching, and C–H bending and ring puckering, respectively (42, 43). As shown in Figure. 5b, the main bands in chitosan powder were 3433 cm^-1^ (O-H stretching), 2875 cm^-1^ (C-H stretching), 1655 cm^-1^ (C=O stretching, amide I representing the structure of N-acetylglucosamine), 1580 cm^-1^ (bending vibrations of the N-H representing N-acetylated residues), 1424 cm^-1^ (N-H stretching, amide II representing glucosa­mine functional groups), 1385 cm^-1^ (NH stretching, amide III), 1159 cm^-1 ^(C-O-C stretching) and 896 cm^-1^ (pyranose ring) (37, 44).

Sodium alginate spectrum (Figure. 5c) showed characteristic bands at 3433 cm^-1^ (OH), 2924 cm^-1 ^(CH), 1630 cm^-1^ (COO- asymmetric), 1416 cm^-1 ^(COO- symmetric), and 1031 cm^-1^ (C-O-C). Similar absorption bands were reported by other authors (37, 45). By comparing the spectrum of blank chitosan-coated alginate microcapsules (Figure 5e) with chitosan (b) and sodium alginate (c), it was found that most specific peaks of chitosan and alginate were present in microcapsules spectrum with some shifts. Stretching vibration of O-H at 3433 cm^-1^ shifted to 3429 cm^-1^. 

Appearance of new peak at 2855 cm^-1 ^and increase of the sharpness of the peak at 1631 cm^−1 ^as a result of COO^-^ groups in alginate and the disappearance of the chitosan amino band at 1580 cm^-1^, suggest the formation of a polyelectrolyte complex between sodium alginate and chitosan (46, 47). A new peal at 1746 cm^-1 ^belonging to COOH is an indicative of acidic condition in which the microcapsules were prepared (37). 

Moreover, some peaks in chitosan spectrum disappeared due to the presence of multi-interactions of polymers like electrostatic and hydrogen bonding (48, 49). Compared to the blank chitosan-coated alginate microcapsules and herbal galactagogue extract spectra, the spectrum of extract-loaded microcapsules (Figure. 5d) is a combination of the two aforementioned spectra and also the appearance of some peaks at 1099, 1061 and 887 cm^-1^ is an indicative of herbal galactagogue extract encapsulation in chitosan-sodium alginate matrix.

*Stability of entrapped flavonoids in microcapsules*

Figure 6 shows the percent loss of flavonoids in microcapsules stored at two different temperatures. As can be seen in Figure 6, although flavonoid loss increased during storage from day 1 to day 120, the loss of flavonoids in the microcapsules was insignificant during the storage. Similarly, in a study by Sansone *et al*. (50), the spray-dried microcapsules containing quercetin were stored at 25 °C for 12 months and after this period, no degradation products or decrease of concentration was observed. Accordingly, Nori *et al*. (51) also pointed out that storage temperatures of 10 and 25 °C had no effect on flavonoid content of microencapsulated propolis extract. The microcapsules stored at 4 °C showed lower percent loss (3.9 ± 0.14%) compared to the samples (6.11 ± 0.21%) stored at 25 °C during 120 days storage. This is in agreement with the results of Abedi *et al*. (52) who reported a higher loss of thymoquinone in microcapsules stored at 20 °C compared to 4 °C. In general, it can be expressed that alginate and chitosan as wall materials are suitable in protection of flavonoids from environmental conditions. 

*Release studies within simulated gastrointestinal conditions*

Figure 7 shows the release of extract from microcapsules in SGF and SIF. Under SGF condition, a fast initial release (18%) during 4 h occurred followed by a slow and continuous release (48.1%) till 24 h. In a study by Dai *et al*. (37), the release of nifedipine from chitosan-coated alginate beads at pH 1.5 was 18% while at pH 6.8, the release increased significantly up to 99%. Similarly, Finotelli *et al*. (53) reported a very fast release of insulin (18%) from alginate/chitosan nanoparticles corresponding to insulin physically entrapped to bead’s external layer. The release of extract from alginate matrix was relatively low due to applying a chitosan coating. The barrier property of chitosan was confirmed in other studies (53-55). At low pH, the carboxyl groups of alginate were protonated and electrostatic repulsion among these groups led to formation of insoluble alginic acid and a reversible shrinkage took place that hindered the release of the core substance (21, 56). Similarly, it was reported that in acidic environment, calcium ions in alginate beads were totally discharged and the carboxyl groups shifted to an un-ionized form (57). In SIF with pH 7, a rapid increase in the release rate was observed up to 80% during 24 h. According to Lacerda *et al*. (21), increase of the pH led to deprotonation of chitosan that attenuated the extent of the interactions inside the microparticle and the ionization of the sodium alginate carboxyl groups. Anal *et al*. (58) reported a negligible release of bovine serum albumin from chitosan-alginate beads in SGF medium (pH 1.2), but in SIF medium (pH 7.5), the release was faster. It was expressed that high affinity of phosphate ions present in intestinal fluid for Ca^+2^ induced disruption of calcium-alginate gel matrix.

## Conclusion

Encapsulation of herbal galactagogue extract in chitosan-coated alginate microcapsules using emulsification/external gelation method in optimum condition (sodium alginate concentration of 1.49%, extract concentration of 1.58% and CaCl_2_ concentration of 0.84%) led to preparation of spherical microcapsules with mean diameter of 123 μm and EE% of 77.97%. FTIR analysis revealed successful extract loading in alginate microcapsules. The *in-vitro* release studies demonstrated a controlled release of extract in SGF and SIF. Based on the results obtained and physicochemical properties of chitosan-coated microcapsules, it can be concluded that these microcapsules can be incorporated into food matrices with neutral pH. 
